# Ethnic Differences in Clinical Manifestations of Behçet's Disease: A Retrospective Cohort Study of Turkic and Non‐Turkic Patients

**DOI:** 10.1002/iid3.70568

**Published:** 2026-11-05

**Authors:** Shokufeh Sadeghi, Seyedeh Tahereh Faezi, Mohsen Rokni, Elham Madreseh, Farhad Shahram, Majid Alikhani, Somayeh Motamed, Fereydoun Davatchi

**Affiliations:** ^1^ Rheumatology Research Center Tehran University of Medical Sciences Tehran Iran; ^2^ Department of Immunology University of Social Welfare and Rehabilitation Sciences Tehran Iran

**Keywords:** Behçet's disease, clinical manifestations, ethnicity, HLA‐B5/51, immunogenic findings, Turkic population

## Abstract

**Introduction:**

Behçet's disease (BD) is a multisystem inflammatory vasculitis with clinical heterogeneity that may vary across ethnic groups within the same country. This study aimed to evaluate the influence of ethnicity on the clinical manifestations, immunogenic laboratory findings and disease course of BD by comparing Turkic and non‐Turkic patients in a large Iranian referral cohort.

**Methods:**

In this retrospective cohort study, 2697 patients with BD followed at the BD clinic of Shariati Hospital in Tehran, Iran, from 2007 to 2019 were included.

**Results:**

Patients were stratified by ethnicity into Turkic (*N* = 1037; 38.5%) and non‐Turkic (*N* = 1660; 61.5% [with Persian/Fars, *N* = 980 subgrouping]) groups, and demographic, clinical manifestations, and immunogenic laboratory findings were extracted from medical records. Mean age at BD onset was 25.72 ± 10.02 years in Turkic patients and 24.69 ± 10.23 years in non‐Turkic patients, and males comprised 61.3% and 57.1% of the two groups, respectively. Human leukocyte antigen (HLA)‐B5 positivity was 55.1% in Turkic versus 53.1% in non‐Turkic patients, and HLA‐B51 positivity was 48.7% versus 46.5%, respectively. Most clinical manifestations showed similar prevalence across groups, including oral aphthous ulcers (98.7% vs. 99.0%), genital aphthous ulcers (60.5% vs. 62.0%), uveitis (50.1% vs. 53.2%), and deep vein thrombosis (5.3% vs. 5.1%). Superficial phlebitis was more frequent in Turkic patients (*p* = 0.02), and oral aphthous ulcers occurred earlier in Turkic patients (*p* = 0.023). A three‐class latent class model demonstrated the best fit (BIC = 14,986.58). In multinomial logistic regression analysis, Turkic patients had slightly higher odds ratio of the ocular‐predominant phenotype (OR = 1.10, 95% CI: 0.91–1.33; *p* = 0.320) and slightly lower odds ratio of the multisystem phenotype (OR = 0.94, 95% CI: 0.68–1.30; *p* = 0.692); however, neither association was statistically significant. Female gender was associated with higher odds ratio of the ocular‐predominant phenotype than the mucocutaneous‐predominant phenotype (OR = 1.38, 95% CI: 1.14–1.67; *p* = 0.001).

**Conclusion:**

In this large Iranian BD cohort, Turkic and non‐Turkic patients exhibited broadly similar disease phenotypes and comparable HLA‐B51 frequencies, with small but potentially relevant ethnic differences in the timing of oral ulcers and the frequency of superficial phlebitis. These findings suggest that ethnicity may influence BD phenotype through mechanisms beyond HLA‐B51, possibly involving other immune‐related genetic variants such as tumor necrosis factor (TNF)‐α, endoplasmic reticulum aminopeptidase 1(ERAP1), Interleukin (IL)‐10. Of note, the significant differences observed in Turkic versus non‐Turkic comparisons were not present in the Turkic versus Persian (Fars) comparison, indicating that the findings are not driven by a single ethnic subgroup.

## 1 Introduction

Behçet's disease (BD) is an autoimmune chronic, relapsing, multisystemic inflammatory vasculitis of unknown etiology, capable of affecting blood vessels of all sizes [1]. First described in 1937 by Hulusi Behçet, a Turkish dermatologist, the disease was initially characterized by a triad of recurrent oral and genital aphthous ulcerations and uveitis [2]. BD predominantly affects populations residing along the ancient Silk Road, spanning from the Mediterranean basin through the Middle East to East Asia, with the highest prevalence reported in Turkey (20–420 per 100,000) and Iran (68 per 100,000), the second highest worldwide [3, 4, 5]. The Iran BD registry, one of the largest globally, has documented 7641 cases from 1975 to 2018, revealing oral aphthosis (97.5%), genital aphthosis (64.4%), skin lesions (62.2%), and ocular involvement (55.6%) as the most frequent manifestations. Males are slightly more commonly affected, with a male‐to‐female ratio of approximately 1.3:1 in Iran. Diagnosis remains clinical, as no pathognomonic laboratory test exists; the revised International Criteria for Behçet's Disease (ICBD) achieved a sensitivity of 96.6% in the Iranian cohort [4, 6, 7, 8, 9].

The etiology of BD involves a complex interplay of genetic susceptibility, environmental triggers, and ethnicity. Human leukocyte antigen (HLA)‐B51 is the most strongly associated genetic marker, particularly in Silk Road populations [6, 7, 8]. Beyond HLA‐B51, genome‑wide association studies (GWAS) have identified several non‐MHC susceptibility loci in BD, including interleukin (IL)‐10, IL23R–IL12RB2, and endoplasmic reticulum aminopeptidase 1 (ERAP1), which interacts epistatically with HLA‐B51. These variants are involved in antigen processing, Th17 polarization, and regulatory cytokine networks, suggesting that BD pathogenesis is driven by a complex interplay of adaptive and innate immune pathways. Ethnic differences in the frequency or functional impact of these variants may contribute to phenotypic heterogeneity [10]. Notably, disease phenotype varies across ethnic groups: ocular involvement predominates in Turkish and Iranian populations, whereas gastrointestinal manifestations are more prevalent among East Asian patients [8, 11, 12, 13, 14]. Studies in French multiethnic cohorts have confirmed that ethnicity independently influences disease manifestations and prognosis. Iran, a genetically diverse nation comprising Turkic (Such as Azeri), Persian (Fars), Kurdish, and other ethnic groups, provides a unique setting to investigate intra‐country ethnic variation [5, 11, 12]. Genetic studies in Iranian Azeri (Turkic) have identified tumor necrosis factor‐alpha (TNF‐α) promoter polymorphisms associated with BD susceptibility, suggesting shared genetic features with Turkish populations in Turkey [15, 16]. However, systematic comparisons of clinical phenotype between ethnic subgroups within Iran remain scarce. Given that Iranian Turkic (Azeri) populations are linguistically and genetically closer to the Turkish populations of Turkey which have the world's highest BD prevalence than to other Iranian ethnic groups, we hypothesized that Turkic patients might exhibit distinct clinical features compared to non‐Turkic Iranians. Therefore, a hypothesis‐driven binary comparison Turkic versus non‐Turkic was performed rather than multiple pairwise ethnic comparisons. This study aimed to evaluate the influence of ethnicity on the clinical and para‐clinical manifestations and disease course of BD by comparing Turkic and non‐Turkic patients in a large Iranian referral cohort.

## 2 Methods

### 2.1 Study Design, Setting, and Participants

This retrospective cohort study included 2697 patients with BD followed at the BD clinic of Shariati Hospital, Tehran University of Medical Sciences, Tehran, Iran, from March 2007 to March 2019. BD was diagnosed/classified using the ICBD criteria [17]. Patients were excluded if they had only a single clinic visit, an alternative diagnosis established during follow‐up, < 2 years of follow‐up, or incomplete medical records for key study variables. Demographic data at BD diagnosis were recorded.

### 2.2 Ethnicity Definition and Grouping

Patients were stratified a priori into two categories: a “Turkic” group (Azeri) and a “Non‐Turkic” group (with Persian/Fars subgrouping) comprising other Iranian ethnicities including Fars, Kurdish, Lor, Arab, or other. Ethnicity was based on self‐report and confirmed, when documented, by familial origin information, including parental ancestry and native language, to reduce misclassification.

### 2.3 Data Collection and Definitions

Medical records from relevant clinic visits were reviewed to extract (I) initial symptoms and clinical findings leading to BD diagnosis and (II) the presence of all recorded clinical manifestations during follow‐up. The date of first manifestation (symptom onset) was defined as the patient‐reported onset date as documented in the record, and the date of diagnosis was operationally defined as the date of the first visit to the BD clinic. Relevant laboratory data including HLA‐B5 and HLA‐B51, when available, were extracted. All BD diagnoses were confirmed by an experienced rheumatologist, and ocular involvement was diagnosed and documented by an ophthalmologist.

### 2.4 Immunological Assessment and HLA Typing

HLA‐B5 and HLA‐B51 data were extracted from the medical records of patients diagnosed between March 2007 and March 2019. During this period, HLA typing was performed using polymerase chain reaction with sequence‐specific primers (PCR‐SSP), the standard molecular method for HLA genotyping. In some cases, particularly in the early years of the study period, serologic methods including complement‐dependent lymphocytotoxicity were used for HLA‐B5 detection. Regardless of the method, HLA‐B5 positivity was defined as the presence of either HLA‐B51 or HLA‐B52, and HLA‐B51 was detected using allele‐specific primers. The high concordance between HLA‐B5 and HLA‐B51 positivity rates (53.9% vs. 47.4% overall) supports the internal consistency of the data across different testing methods and laboratories.

### 2.5 Ethics Statement

This study was approved by the Tehran University of Medical Sciences Institutional Review Board with Ethics/Approval Code: IR.TUMS.MEDICINE.REC.1397.162, which waived informed consent because this was a retrospective review of existing clinical records, and it was conducted in accordance with the World Medical Association Declaration of Helsinki [18].

### 2.6 Statistical Analysis

Continuous variables are presented as mean ± standard deviation (±SD), and categorical variables as counts and percentages. Statistical analyses were performed using IBM SPSS Statistics for Windows (version 23.0). Because a substantial proportion of patients had already developed clinical manifestations before the index date, time‐to‐event modeling such as Cox regression was not used; instead, comparisons relied on group‐based analyses of recorded time intervals and manifestation frequencies. Ethnic‐group comparisons used independent‐samples *t*‐tests for continuous variables and *χ* ^2^ tests for categorical variables with Fisher's exact test when expected cell counts were small. Latent class analysis (LCA) was performed using the poLCA package in R. Models with II–VII classes were fitted using 50 random starts (nrep = 50) to avoid local maxima. Model selection was based on Bayesian Information Criterion (BIC), entropy, and interpretability. Multinomial logistic regression was used to examine the association between latent classes and clinical covariates. Results are presented as odds ratios (ORs) with 95% confidence intervals (CIs). Statistical significance was defined as a two‐sided *p* value ≤ 0.05.

## 3 Results

### 3.1 Study Population

The study included 2697 patients with BD including 1037 (38.5%) of Turkic (Azeri) ethnicity and 1660 (61.5%) from other Iranian ethnic backgrounds (non‐Turkic group). Also, the Persian subgroup (Fars) was adequately represented in our cohort (980 Persian (Fars) vs. 1037 Turkic patients), while other ethnicities accounted for only 22.9% of the study population (see Tables 1 and 2). This enables direct assessment of whether the observed significant differences are driven by the Persian subgroup or by other ethnic groups within the non‐Turkic category.

The mean age at BD onset was 25.72 ± 10.02 years in Turkic patients and 24.69 ± 10.23 years in non‐Turkic patients. Males comprised 61.3% (635/1037) of the Turkic group and 57.1% (901/1660) of the non‐Turkic group, corresponding to male‐to‐female ratios of 1.59:1 and 1.34:1, respectively.

### 3.2 Immunogenic Laboratory Findings

HLA‐B5 testing was available for 2615 patients; 1409 (53.9%) were HLA‐B5 positive. Among Turkic patients, 571/1037 (55.1%) were HLA‐B5 positive compared with 838/1578 (53.1%) in the non‐Turkic group, with no significant between‐group difference (*p* = 0.336).

HLA‐B51 testing was available for 2614 patients, of whom 1238 (47.4%) were positive. HLA‐B51 positivity was 48.7% (505/1036) in Turkic patients and 46.5% (733/1578) in non‐Turkic patients, with no significant difference (*p* = 0.262).

### 3.3 Clinical Manifestations

Table 1 summarizes the prevalence of BD clinical manifestations by ethnicity. Mucosal involvement was almost universal, with oral aphthous ulcers in 98.7% of Turkic patients and 99.0% of non‐Turkic patients (*p* = 0.588), and genital aphthous ulcers in 60.5% versus 62.0%, respectively (*p* = 0.383). Skin manifestations were also similar between groups, including pseudofolliculitis (29.6% vs. 30.2%, *p* = 0.772) and erythema nodosum (21.5% vs. 21.0%, *p* = 0.737).

**Table 1:** Prevalence of clinical manifestations of Behçet's disease by ethnicity (Turkic vs. non‐Turkic and Persian [Fars]).

Signs and symptoms	Ethnicity (*N* %)			Turkic *p* value vs.	
	Turkic *N* = 1037	Non‐Turkic *N* = 1660	Persian (Fars) *N* = 980	Non‐Turkic	Persian (Fars)
*Mucosal involvement*	1028 (99.1%)	1564 (99.1%)	969 (98.9%)	0.959	0.655
Oral aphthous ulcers	1024 (98.7%)	1562 (99.0%)	967 (98.7%)	0.588	0.320
Genital aphthous ulcers	627 (60.5%)	978 (62.0%)	595 (60.7%)	0.383	0.854
*Skin involvement*	490 (47.3%)	714 (45.2%)	435 (44.4%)	0.314	0.211
Pseudofolliculitis	307 (29.6%)	476 (30.2%)	285 (29.1%)	0.772	0.807
Erythema nodosum	223 (21.5%)	331 (21.0%)	204 (20.8%)	0.737	0.703
Other skin lesions	105 (10.1%)	133 (8.4%)	83 (8.5%)	0.138	0.220
*Ocular lesions*	597 (57.6%)	929 (58.9%)	580 (59.2%)	0.509	0.470
Uveitis	520 (50.1%)	839 (53.2%)	522 (53.3%)	0.130	0.167
Anterior uveitis	403 (38.9%)	632 (40.1%)	390 (39.8%)	0.556	0.682
Posterior uveitis	465 (44.8%)	735 (46.6%)	457 (46.6%)	0.395	0.447
Retinal vasculitis	353 (34.0%)	546 (34.6%)	328 (33.5%)	0.781	0.778
Cataracts	302 (29.1%)	405 (25.7%)	253 (25.8%)	0.050*	0.100
Conjunctivitis	39 (3.8%)	64 (4.1%)	43 (4.4%)	0.708	0.479
*Vascular lesions*	79 (7.6%)	96 (6.1%)	56 (5.7%)	0.125	0.087
DVT (phlebitis)	55 (5.3%)	80 (5.1%)	48 (4.9%)	0.791	0.687
Arterial thrombosis	4 (0.4%)	5 (0.3%)	2 (0.2%)	0.769	0.688
Vascular aneurysm	5 (0.5%)	5 (0.3%)	4 (0.4%)	0.503	1.000
Superficial phlebitis	24 (2.3%)	18 (1.1%)	12 (1.2%)	0.020*	0.091
*Joint symptoms*	368 (35.5%)	568 (36.0%)	353 (36.0%)	0.791	0.816
Arthralgia	181 (17.5%)	276 (17.5%)	170 (17.3%)	0.990	0.953
Monoarthritis	104 (10.0%)	131 (8.3%)	82 (8.4%)	0.129	0.218
Oligoarthritis	127 (12.2%)	235 (14.9%)	146 (14.9%)	0.057	0.090
Spondyloarthropathy	23 (2.2%)	32 (2.0%)	21 (2.1%)	0.738	1.000
*Gastrointestinal symptoms*	34 (3.3%)	65 (4.1%)	39 (4.0%)	0.271	0.407
Gastroduodenitis	6 (0.6%)	8 (0.5%)	6 (0.6%)	0.805	1.000
Peptic ulcer	3 (0.3%)	6 (0.4%)	6 (0.6%)	0.699	0.331
Rectorrhagia	6 (0.6%)	9 (0.6%)	6 (0.6%)	0.977	1.000
*Neurologic symptoms*	67 (6.5%)	128 (8.1%)	86 (8.8%)	0.116	0.053
*Pulmonary symptoms*	9 (0.9%)	8 (0.5%)	4 (0.4%)	0.262	0.268
*Cardiovascular symptoms*	4 (0.4%)	5 (0.3%)	4 (0.4%)	0.747	1.000

*Note:* Data are presented as *N* (%).

* *p* < 0.05.

Also, ocular involvement did not differ significantly overall (57.6% vs. 58.9%, *p* = 0.509), including uveitis (50.1% vs. 53.2%, *p* = 0.130). Vascular involvement was comparable between groups (7.6% vs. 6.1%, *p* = 0.125), including deep vein thrombosis (5.3% vs. 5.1%, *p* = 0.791). Cataract was marginally more frequent in Turkic patients (29.1% vs. 25.7%, *p* = 0.050), whereas superficial phlebitis was significantly more common in the Turkic group (2.3% vs. 1.1%, *p* = 0.020). Several manifestations showed borderline trends, including oligoarthritis, which was more frequent in non‐Turkic patients (14.9% vs. 12.2%, *p* = 0.057).

### 3.4 Timing of Manifestations

Table 2 presents the mean interval (years ± SD) from BD diagnosis on the first BD‐clinic visit to the onset of manifestations. Oral aphthous ulcers developed earlier in Turkic patients (0.23 ± 1.29 vs. 0.37 ± 1.81 years, *p* = 0.023), while superficial phlebitis occurred significantly later in non‐Turkic patients (10.73 ± 8.23 vs. 5.53 ± 5.93 years, *p* = 0.022). Other manifestations showed no significant ethnic differences in timing, including genital aphthous ulcers with 5.01 ± 6.10 versus 4.94 ± 5.89 years, *p* = 0.843 and posterior uveitis with 5.90 ± 6.57 versus 6.14 ± 6.96 years, *p* = 0.555.

**Table 2:** Association between ethnicity and time from BD diagnosis to onset of manifestations (Turkic vs. non‐Turkic and Persian [Fars]).

Signs and symptoms	Ethnicity (mean years ± SD)			Turkic *p* value vs.	
	Turkic *N* = 1037	Non‐Turkic *N* = 1660	Persian (Fars) *N* = 980	Non‐Turkic	Persian (Fars)
*Mucosal involvement*	0.21 ± 1.18	0.28 ± 1.60	0.34 ± 1.75	0.175	0.104
Oral aphthous ulcers	0.23 ± 1.29	0.37 ± 1.81	0.46 ± 2.10	0.023*	0.132
Genital aphthous ulcers	5.01 ± 6.10	4.94 ± 5.89	5.19 ± 6.01	0.843	0.584
*Skin involvement*	5.64 ± 6.57	5.47 ± 7.13	5.78 ± 6.50	0.662	0.590
Pseudofolliculitis	5.48 ± 6.73	5.53 ± 7.24	5.99 ± 6.41	0.924	0.706
Erythema nodosum	6.20 ± 6.72	6.04 ± 7.03	6.44 ± 7.19	0.792	0.651
Other skin lesions	6.67 ± 6.69	6.40 ± 6.95	6.94 ± 7.52	0.769	0.523
*Ocular lesions*	6.43 ± 7.07	6.24 ± 6.93	6.62 ± 7.22	0.612	0.449
Uveitis	5.75 ± 6.58	5.99 ± 6.86	6.27 ± 7.12	0.529	0.110
Anterior uveitis	5.65 ± 6.44	6.00 ± 6.99	6.09 ± 6.99	0.416	0.173
Posterior uveitis	5.90 ± 6.57	6.14 ± 6.96	6.37 ± 7.11	0.555	0.555
Retinal vasculitis	6.49 ± 6.60	6.78 ± 6.78	6.95 ± 6.73	0.530	0.530
Cataracts	9.07 ± 7.33	8.70 ± 7.11	9.31 ± 7.47	0.497	0.676
Conjunctivitis	6.99 ± 5.70	8.17 ± 7.56	8.89 ± 8.56	0.379	0.151
*Vascular lesions*	7.00 ± 8.20	6.64 ± 6.45	7.53 ± 6.89	0.747	0.711
DVT (phlebitis)	8.49 ± 8.87	6.36 ± 5.94	7.29 ± 6.39	0.124	0.102
Arterial thrombosis	1.50 ± 1.00	7.99 ± 0.28	7.01 ± 2.82	0.232	0.128
Vascular aneurysm	4.40 ± 4.40	5.17 ± 3.25	5.46 ± 3.68	0.762	0.762
Superficial phlebitis	5.53 ± 5.93	10.73 ± 8.23	11.67 ± 8.30	0.022*	0.195
*Joint symptoms*	7.48 ± 7.33	7.06 ± 7.00	7.59 ± 7.17	0.371	0.815
Arthralgia	8.43 ± 7.01	8.65 ± 7.66	9.21 ± 8.10	0.764	0.716
Monoarthritis	8.07 ± 8.08	8.66 ± 7.69	9.42 ± 7.40	0.536	0.347
Oligoarthritis	6.81 ± 7.43	6.09 ± 6.87	6.97 ± 7.08	0.351	0.283
Spondyloarthropathy	8.54 ± 8.02	9.30 ± 7.31	9.71 ± 8.12	0.715	0.657
*Gastrointestinal symptoms*	7.73 ± 6.67	7.82 ± 7.38	8.90 ± 8.46	0.949	0.438
Gastroduodenitis	10.48 ± 8.82	8.01 ± 5.10	9.51 ± 4.97	0.518	0.525
Peptic ulcer	8.67 ± 13.33	10.84 ± 13.80	10.84 ± 13.80	0.829	0.912
Rectorrhagia	6.11 ± 4.93	7.96 ± 5.80	8.67 ± 5.01	0.533	0.233
*Neurologic symptoms*	7.03 ± 6.02	7.81 ± 6.74	8.13 ± 6.42	0.434	0.525
*Pulmonary symptoms*	9.56 ± 8.89	6.88 ± 5.57	6.25 ± 6.76	0.475	0.438
*Cardiovascular symptoms*	9.85 ± 8.71	11.04 ± 6.32	13.49 ± 4.17	0.807	0.478

*Note:* Data are presented as mean years ± SD.

* *p* < 0.05.

### 3.5 Latent Class Analysis and Association With Turkic Ethnicity

Model fit indices for latent class solutions ranging from II to VII classes are presented in Table 3. Although the two‐class solution demonstrated the highest entropy (0.939) and the lowest classification error (0.01), a three‐class model showed the best fit based on the lowest BIC (BIC = 14,986.58) and interpretability. Models with more than three classes did not improve model fit and showed increased complexity without meaningful gains in interpretability or classification performance. Therefore, the three‐class solution was selected as the optimal model for subsequent analysis, because it provided the lowest BIC and clinically interpretable phenotypic profiles. The classes included: Class I (26.0%), Class II (13.0%), and Class III (61.0%). Class I was characterized by ocular‐predominant involvement, Class II by multisystem severe involvement, and Class III by mucocutaneous‐predominant disease.

**Table 3:** Model fit indices and classification performance for latent class solutions (II–VII classes) in the overall cohort and by ethnicity (Turkic and non‐Turkic).

Classes number	BIC	Log‐likelihood	Entropy	No. of parameters	df	*p*	Classification error
*Total ethnicity*							
II	15,004.46	−7435.424	0.939	17	238	0.017*	0.01
III	14,986.58	−7391.113	0.618	26	229	0.934	0.15
IV	15,016.36	−7370.635	0.735	35	220	0.999	0.14
V	15,061.27	−7357.717	0.673	44	211	0.999	0.17
VI	15,111.87	−7347.650	0.688	53	202	0.999	0.20
VII	15,166.18	−7339.437	0.715	62	193	0.999	0.20
*Non‐Turkic ethnicity*							
II	9080.405	−4477.690	0.837	17	238	0.994	0.03
III	9097.837	−4453.312	0.679	26	229	0.999	0.13
IV	9133.210	−4437.904	0.583	35	220	0.999	0.26
V	9181.371	−4428.890	0.646	44	211	0.999	0.24
VI	9234.415	−4422.317	0.636	53	202	0.999	0.27
VII	9290.561	−4417.295	0.669	62	193	0.999	0.28
*Turkic ethnicity*							
II	6011.215	−2946.657	0.950	17	238	0.994	0.01
III	6018.302	−2918.991	0.649	26	229	0.999	0.15
IV	6050.284	−2903.773	0.747	35	220	0.999	016
V	6097.587	−2896.216	0.781	44	211	0.999	0.15
VI	6149.985	−2891.205	0.780	53	202	0.999	0.18
VII	6203.536	−2886.772	0.786	62	193	0.999	0.20

Abbreviation: BIC, Bayesian Information Criterion.

* *p* < 0.05.

The latent classes were characterized based on the conditional probabilities of clinical manifestations (Figure S1). Class I was defined by a very high probability of ocular involvement (1.000) combined with low probabilities across other organ systems, including skin/mucocutaneous (0.185), vascular (0.022), and gastrointestinal involvement (0.016), suggesting an ocular‐dominant phenotype with limited systemic involvement. Class II was characterized by a high probability of skin/mucocutaneous involvement (0.890) together with moderate probabilities of joint (0.572) and renal involvement (0.368), indicating a skin‐predominant multisystem inflammatory phenotype with intermediate systemic spread. Class III demonstrated a very high probability of skin/mucocutaneous involvement (0.979) with low probabilities in all other organ systems such as ocular 0.387, vascular 0.055, renal 0.078, suggesting a skin/mucocutaneous‐predominant and largely systemically limited phenotype.

Overall, the latent class structure revealed three clinically distinct phenotypes: an ocular‐dominant class (Class I), a multisystem inflammatory class with skin predominance (Class II), and a skin/mucocutaneous‐limited phenotype (Class III).

In multinomial logistic regression analysis, with the mucocutaneous‐predominant phenotype (Class III) as the reference category, neither the unadjusted nor the adjusted model (adjusted for age at onset, sex, and disease duration) showed a significant association between Turkic ethnicity and phenotype class membership. Compared with non‐Turkic patients, Turkic patients had slightly higher OR of the ocular‐predominant phenotype (adjusted OR = 1.10, 95% CI: 0.91–1.33; *p* = 0.320) and slightly lower OR of the multisystem phenotype (adjusted OR = 0.94, 95% CI: 0.68–1.30; *p* = 0.692); however, neither association was statistically significant. Female sex was associated with higher OR of the ocular‐predominant phenotype than the mucocutaneous‐predominant phenotype (adjusted OR = 1.38, 95% CI: 1.14–1.67; *p* = 0.001); see in Table 4.

**Table 4:** Multinomial logistic regression of Turkic ethnicity across phenotype classes.

Classes	Variable	Unadjusted OR (95% CI)	*p*	Adjusted OR (95% CI)^a^	*p*
I	Turkic ethnicity	1.09 (0.90–1.32)	0.369	1.10 (0.91–1.33)	0.320
I	Female gender	—	—	1.38 (1.14–1.67)	0.001*
II	Turkic ethnicity	0.95 (0.69–1.32)	0.763	0.94 (0.68–1.30)	0.692

*Note:* Class III (skin/mucocutaneous‐limited phenotype) was used as the reference category. Values are presented as odds ratios (OR) with 95% confidence intervals (CI).

^a^ The adjusted model includes age at onset, gender, and disease duration.

* *p* < 0.05.

To further investigate whether phenotypic clustering differed by ethnicity, separate LCAs were performed in Turkic and non‐Turkic patients. Although the two‐class solution yielded the lowest BIC in both ethnic subgroups, the three‐class solution was additionally examined to facilitate direct comparison with the overall cohort and to evaluate the consistency of phenotypic patterns across ethnicities.

Three clinically interpretable phenotypes were identified in both ethnic groups: an ocular‐predominant phenotype, a multisystem phenotype, and a mucocutaneous‐predominant phenotype. The ocular‐predominant phenotype was characterized by a very high probability of ocular involvement (1.00 in both groups) with minimal involvement of other organ systems. Likewise, the mucocutaneous‐predominant phenotype demonstrated a high probability of mucocutaneous manifestations (0.97 in non‐Turkic and 0.98 in Turkic patients) with limited systemic involvement. The multisystem phenotype showed concurrent involvement of multiple organ systems in both ethnic groups; however, minor differences were observed in the conditional probabilities of individual manifestations. Compared with non‐Turkic patients, Turkic patients with the multisystem phenotype exhibited higher probabilities of ocular (0.98 vs. 0.69) and articular involvement (0.70 vs. 0.50), whereas non‐Turkic patients showed higher probabilities of mucocutaneous (0.92 vs. 0.71), renal (0.61 vs. 0.19), neurologic (0.31 vs. 0.15), vascular (0.25 vs. 0.13), and gastrointestinal involvement (0.18 vs. > 0.00). Overall, despite these quantitative differences, the overall phenotypic patterns were broadly comparable between the two ethnic groups; see in Figure 1 and Table 3.

**Figure 1:**
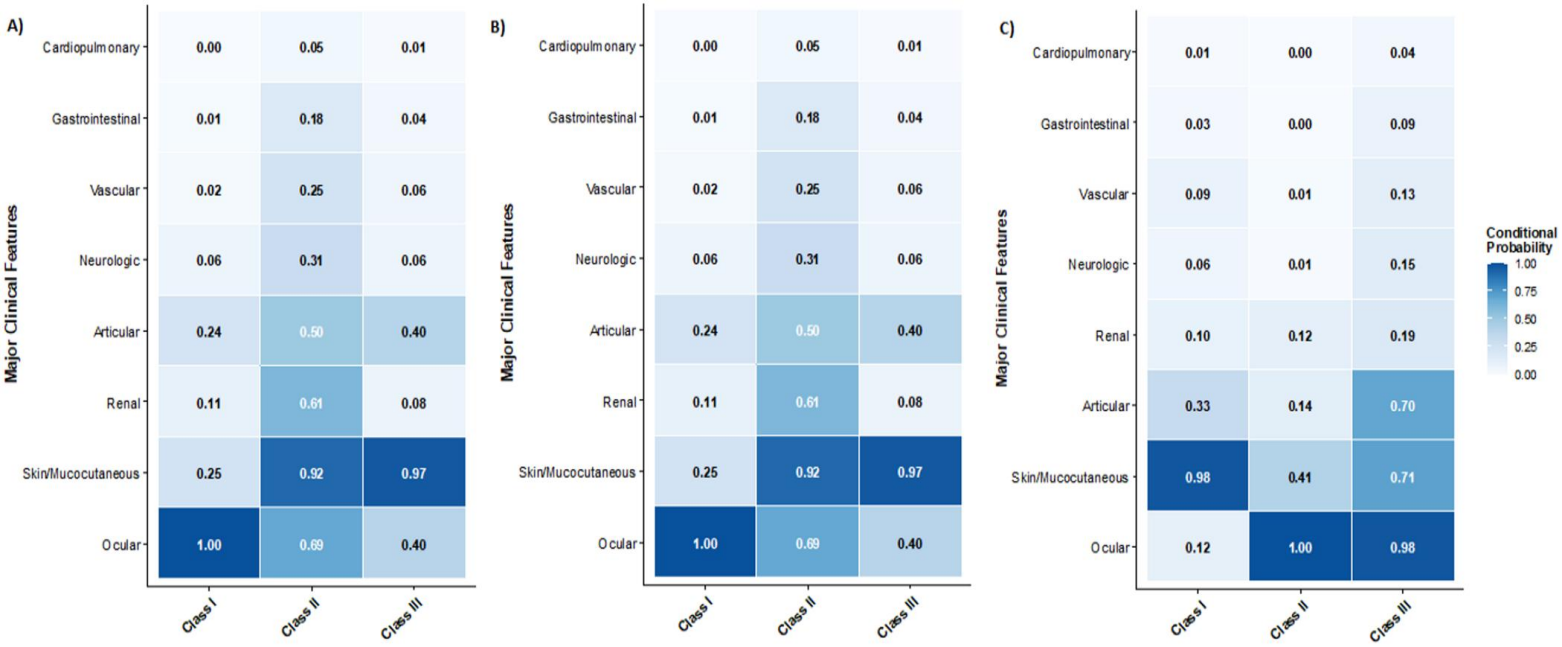
Heatmaps of the conditional probabilities of major clinical manifestations across latent classes identified by latent class analysis (LCA). Each cell represents the estimated conditional probability of the presence of a given clinical manifestation within a latent class. Darker colors indicate higher conditional probabilities, and the numerical values represent the estimated probabilities. The latent classes correspond to clinically interpretable phenotypes based on patterns of organ involvement. (A) Overall cohort, (B) non‐Turkic patients, and (C) Turkic patients. Because latent class labels are arbitrary, class numbering is specific to each analysis and should not be interpreted as indicating direct correspondence between classes across the overall cohort and the ethnicity‐specific subgroup analyses.

## 4 Discussion

This large, single‐center comparative cohort of Iranian patients with BD stratified by Turkic versus non‐Turkic ethnicity highlights a predominantly shared clinical phenotype with a small number of statistically significant between‐group differences. Although HLA‐B51 prevalence was similar between groups (48.7% vs. 46.5%; *p* = 0.262), Turkic patients developed oral aphthous ulcers slightly earlier (0.23 vs. 0.37 years from diagnosis; *p* = 0.023) and had a higher frequency of superficial phlebitis (2.3% vs. 1.1%; *p* = 0.020).

HLA‐B51 remains the strongest and most consistently replicated genetic risk factor for BD across “Silk Road” populations, yet it does not fully explain inter‐individual and inter‐population heterogeneity in organ involvement or disease course [6, 7, 8]. In keeping with this, GWAS have identified additional susceptibility loci including IL‐10 and the IL‐23R–IL‐12RB2 region, supporting the concept that non‐HLA immune pathways may modify BD expression even when HLA‐B51 frequencies are comparable between groups [10]. Moreover, ERAP1 has been implicated in BD susceptibility with evidence of interaction with HLA‐B51, offering a plausible mechanism by which ethnicity‐associated genetic architecture could influence selected manifestations without changing overall HLA‐B51 prevalence [19]. The comparable frequency of HLA‐B51 between Turkic and non‑Turkic patients does not exclude the possibility that other immune‐related polymorphisms differ between these populations. For example, TNF‐α promoter polymorphisms, which have been associated with BD susceptibility in Iranian Azeri Turkics, or ERAP1 haplotypes, which show population‐specific diversity, could influence vascular inflammation without altering HLA‐B51 carriage [15]. Future immunogenetic studies are needed to dissect these layers of regulation.

The near‐universal mucosal involvement in our cohort, contrasted with the low frequency of gastrointestinal manifestations (3.3%–4.1%), reinforces the concept that BD has a stable “core” mucocutaneous phenotype in Iran, while the prevalence of gastrointestinal disease varies substantially by geography and ethnicity [3, 4, 5, 11, 12, 13, 20]. Prior work from Iran has similarly shown high rates of oral aphthosis and genital aphthosis in large registry analyses, supporting the generalizability of mucosal predominance in Iranian BD [11, 12]. Conversely, gastrointestinal BD has been reported more commonly in East Asian cohorts, consistent with the marked geographic variation described in contemporary reviews [13, 20].

The higher prevalence of superficial phlebitis in Turkic patients and its later mean onset among non‐Turkic patients may indicate modest ethnic differences in the vascular inflammatory phenotype, but these findings should be interpreted in light of the low absolute event rates and the potential for diagnostic overlap between superficial thrombophlebitis and erythema nodosum‐like lesions in routine clinical documentation [4, 20]. The higher frequency of superficial phlebitis in Turkic patients, while statistically significant, was based on low absolute event rates (2.3% vs. 1.1%). Therefore, this finding should be considered hypothesis‐generating and requires replication in independent, larger cohorts before definitive conclusions can be drawn. Although statistically significant, the finding was based on a small number of events and therefore should be interpreted cautiously.

Interestingly, when Turkic patients were compared directly with Persian (Fars) patients, none of the previously significant differences remained statistically significant. This suggests that the significant findings observed in the Turkic versus non‐Turkic comparison are not attributable solely to the Persian subgroup, but may reflect the broader ethnic composition of the non‐Turkic group. These findings support the use of a composite non‐Turkic comparison group.

Cataract was marginally more frequent in Turkic patients (29.1% vs. 25.7%, *p* = 0.05) that may reflect differences in cumulative ocular inflammation and/or corticosteroid exposure, since cataract is a well‐recognized complication in Behçet uveitis and chronic uveitis management [21]. As shown in Table 2, cataract developed after a mean interval of 9.07 ± 7.33 years in Turkic patients and 8.70 ± 7.11 years in non‑Turkic patients (*p* = 0.497). This is considerably later than the onset of oral aphthous ulcers (0.23–0.37 years; Table 2), confirming that cataract is a secondary complication of BD, typically resulting from chronic uveitis or long‑term corticosteroid use, rather than an initial manifestation. The borderline higher frequency in Turkic patients may reflect differences in cumulative uveitis activity, duration of follow‐up, or steroid exposure. However, because we did not model treatment intensity, steroid burden, or uveitis severity longitudinally, this observation should be considered hypothesis‐generating rather than causal. Beside, cataract is typically a secondary complication of chronic uveitis and its therapy such as corticosteroid consumption rather than a direct disease manifestation. We did not have longitudinal data on uveitis severity or cumulative steroid exposure, which may have contributed to the borderline higher frequency observed in Turkic patients. Future studies should adjust for these treatment‐related factors.

The LCA revealed that the three distinct clinical phenotypes (ocular, mucocutaneous, and multisystem) were present in both ethnic groups with broadly similar conditional probability patterns, further supporting the presence of a shared disease phenotype across Iranian ethnicities. This suggests that ethnicity does not fundamentally alter the clinical structure of BD in Iran, and that the observed minor differences in individual symptom frequencies do not translate into distinct phenotypic clustering patterns.

Our findings align with evidence from multiethnic cohorts showing that ethnicity can be associated with differences in BD manifestations and outcomes, supporting the need to consider ethnicity alongside clinical factors when monitoring for specific organ complications [14]. At the same time, the overall similarity in most manifestations between groups suggests that established BD diagnostic and management approaches remain broadly applicable across Iranian ethnicities, with selective attention to vascular signs and symptom chronology where differences were observed [4, 20]. Given the exploratory nature of the analyses and the large number of comparisons performed, statistically significant findings such as superficial phlebitis and oral aphthous ulcer timing should be interpreted cautiously and considered hypothesis‐generating until replicated in independent cohorts.

### 4.1 Study Limitations

This study has several limitations. First, the non‑Turkic group included heterogeneous ethnicities mainly Persian, Kurdish, and others, and the sample sizes of individual subgroups including Persian alone or Kurdish alone were insufficient for statistically reliable one‐on‐one comparisons with the Turkic group. The non‐Turkic group comprised several ethnic backgrounds (Persian, Kurdish, and others), which may have reduced the ability to detect ethnicity‐specific associations. Second, the absolute frequency of superficial phlebitis was low (2.3% vs. 1.1%), which limits the generalizability of this finding and increases the risk of type I error. Third, we did not have access to genetic data beyond HLA‐B51 such as TNF‐α promoter polymorphisms, ERAP1 variants, which could have helped explain the biological basis of ethnic differences in vascular manifestations. Fourth, the retrospective design and reliance on patient‐reported onset dates recorded in medical charts may introduce recall and documentation bias. Fifth, defining the “diagnosis date” as the first visit to our BD clinic may not capture earlier diagnoses made elsewhere. Sixth, HLA testing was not available for all patients, and the large number of statistical comparisons increases the risk of chance findings, particularly for borderline *p* values. Finally, although our center is the national referral center for BD in Iran, receiving patients from across the country, this remains a single‐country study. Therefore, generalizability to other populations with different genetic backgrounds may be limited. No genetic, molecular, or functional analyses were available to verify the biological mechanisms underlying the observed ethnic differences. However, by presenting both Turkic versus non‐Turkic and Turkic versus Persian comparisons in Tables 1 and 2, we have now directly addressed the concern about ethnic heterogeneity within the non‐Turkic group.

### 4.2 Future Research Directions

Future studies should address the limitations of the current work. First, large, multicenter, international cohorts with more genetically distant populations such as Turkish, Japanese, Mediterranean, and European are needed to better elucidate ethnic influences on BD phenotype beyond the similarities observed between Turkic and non‐Turkic Iranians. Second, prospective studies with genotyping of TNF‐α promoter polymorphisms and other inflammatory pathway genes such as ERAP1, IL‐10, IL23R are required to understand the biological basis of ethnic differences in vascular manifestations such as superficial phlebitis. Third, functional in vitro studies including clot formation assays or cytokine production assays using patient‐derived samples are warranted to confirm whether genetic polymorphisms translate into functional and clinical differences. Also, treatment exposure data such as corticosteroid burden, immunosuppressive therapy should be incorporated in future studies to assess whether ethnic differences in complications such as cataract are driven by treatment intensity rather than disease activity alone. Finally, prospective, standardized phenotyping with predefined data collection instruments will help reduce recall and documentation bias.

## 5 Conclusion

In this large retrospective cohort from a national BD referral center in Iran, Turkic and non‐Turkic patients showed broadly similar clinical phenotypes and comparable HLA‐B51 prevalence, supporting the presence of a shared “core” BD presentation across Iranian ethnicities. Nevertheless, modest ethnicity‐associated differences were observed, including earlier onset of oral aphthous ulcers in Turkic patients and a higher frequency of superficial phlebitis, while most other mucocutaneous, ocular, and major vascular manifestations were not significantly different between groups. These findings suggest that, within Iran, ethnicity may influence selected aspects of BD chronology and vascular expression beyond HLA‐B51 carriage alone and may help clinicians tailor monitoring for specific complications. From an immunological perspective, our findings reinforce that BD is a polygenic disease where ethnicity‑associated genetic variation beyond HLA‐B51 such as in cytokine pathways or antigen processing machinery may improve clinical expression. Prospective multicenter studies incorporating standardized phenotyping and treatment/exposure data are warranted to confirm these small differences and clarify underlying mechanisms.

## Author Contributions

Shokufeh Sadeghi conducted and oversaw the study. Mohsen Rokni drafted, edited, and revised the manuscript and organized the tables/figures. Farhad Shahram, Fereydoun Davatchi, Shokufeh Sadeghi, Majid Alikhani, Somayeh Motamed, and Seyedeh Tahereh Faezi contributed to disease diagnosis. Elham Madreseh performed statistical analysis. Seyedeh Tahereh Faezi formulated and designed the study, as well as edited the draft. All authors critically reviewed and endorsed the final manuscript.

## Ethics Statement

All procedures performed in studies involving human participants were by the ethical standards of the institutional and/or national research committee and with the 1964 Helsinki Declaration and its later amendments or comparable ethical standards. The study protocol was approved by the Ethics Committee of the TUMS (Ethical Code: IR.TUMS.MEDICINE.REC.1397.162). During the preparation of this work, the authors used an AI‐based language tool solely for spelling correction, grammar checking, and minor text improvement. After using this tool, the authors reviewed and edited the content as needed and take full responsibility for the final published version.

## Consent

Informed consent was waived because this was a retrospective review of existing clinical records.

## Conflicts of Interest

The authors declare no conflicts of interest.

## Supporting information


Supporting File

## Data Availability

The data that support the findings of this study are available from the corresponding author upon reasonable request.

## References

[1] M. Rokni , M. Khomeijani‐Farahani , T. Soltani , A. Jamshidi , M. Mahmoudi , and E. Farhadi , “Understanding the Pleiotropic Effects of CXCL10/IP‐10 in the Immunopathogenesis of Inflammatory Rheumatic Diseases: Implications for Better Understanding Disease Mechanisms,” International Immunopharmacology 153 (2025): 114456. 10.1016/j.intimp.2025.114456 40121742

[2] A. Greco , A. De Virgilio , M. Ralli , et al., “Behçet's Disease: New Insights Into Pathophysiology, Clinical Features and Treatment Options,” Autoimmunity Reviews 17, no. 6 (June 2018): 567–575, 10.1016/j.autrev.2017.12.006. 29631062

[3] H. Keino and A. A. Okada , “Behcet's Disease: Global Epidemiology of an Old Silk Road Disease,” British Journal of Ophthalmology 91, no. 12 (2007): 1573–1574. 10.1136/bjo.2007.124875 PMC2095534 18024803

[4] F. Davatchi , C. Chams‐Davatchi , H. Shams , et al., “Behcet's Disease: Epidemiology, Clinical Manifestations, and Diagnosis,” Expert Review of Clinical Immunology 13, no. 1 (2017): 57–65. 10.1080/1744666X.2016.1205486 27351485

[5] F. Davatchi , A. R. Jamshidi , A. T. Banihashemi , et al., “Prevalence of Behcet's Disease in Iran: A WHO‐ILAR COPCORD Stage I Study,” APLAR Journal of Rheumatology 10, no. 3 (2007): 239–243.

[6] M. de Menthon , M. P. Lavalley , C. Maldini , L. Guillevin , and A. Mahr , “HLA–B51/B5 and the Risk of Behçet's Disease: A Systematic Review and Meta‐Analysis of Case–Control Genetic Association Studies,” Arthritis and Rheumatism 61, no. 10 (2009): 1287–1296. 10.1002/art.24642 PMC3867978 19790126

[7] T. I. Kaya , “Genetics of Behçet's Disease,” Pathology Research International 2012, no. 1 (2012): 912589. 10.1155/2012/912589 PMC3195436 22013548

[8] A. Sadeghi , M. Rostami , G. Amraei , et al., “Clinical Manifestations of Behçet's Disease: A Retrospective Cross‐Sectional Study,” Mediterranean Journal of Rheumatology 34, no. 1 (2023): 53–60. 10.31138/mjr.34.1.53 PMC10201098 37223587

[9] S. Sadeghi , S. T. Faezi , H. Haerian , et al., “Clinical Features and Chronology of Clinical Manifestations in Behçet's Disease: A Cohort of 2,615 Patients,” Rheumatology 63, no. 5 (2025): 296–303. 10.5114/reum/202818 PMC12673816 41347105

[10] E. F. Remmers , F. Cosan , Y. Kirino , et al., “Genome‐Wide Association Study Identifies Variants in the MHC Class I, IL10, and IL23R‐IL12RB2 Regions Associated With Behçet's Disease,” Nature Genetics 42, no. 8 (2010): 698–702. 10.1038/ng.625 PMC2923807 20622878

[11] F. Davatchi , F. Shahram , C. Chams‐Davatchi , et al., “Behcet's Disease in Iran: Analysis of 7641 Cases,” Modern Rheumatology 29, no. 6 (2019): 1023–1030. 10.1080/14397595.2018.1558752 30557064

[12] F. Davatchi , C. Chams‐Davatchi , H. Shams , et al., “Adult Behcet's Disease in Iran: Analysis of 6075 Patients,” International Journal of Rheumatic Diseases 19, no. 1 (2016): 95–103. 10.1111/1756-185X.12691 26258691

[13] H. Ideguchi , A. Suda , M. Takeno , et al., “Gastrointestinal Manifestations of Behçet's Disease in Japan: A Study of 43 Patients,” Rheumatology International 34, no. 6 (2014): 851–856. 10.1007/s00296-013-2838-5 23934478

[14] L. Savey , M. Resche‐Rigon , B. Wechsler , et al., “Ethnicity and Association With Disease Manifestations and Mortality in Behçet's Disease,” Orphanet Journal of Rare Diseases 9, no. 1 (2014): 42. 10.1186/1750-1172-9-42 PMC3986678 24674137

[15] M. Bonyadi , Z. Jahanafrooz , M. Esmaeili , et al., “TNF‐α Gene Polymorphisms in Iranian Azeri Turkish Patients With Behcet's Disease,” Rheumatology International 30, no. 2 (2009): 285–289. 10.1007/s00296-009-1134-x 19774383

[16] R. Abdolmohammadi and M. Bonyadi , “Polymorphisms of Promoter Region of TNF‐α Gene in Iranian Azeri Turkish Patients With Behçet's Disease,” Journal of Korean Medical Science 32, no. 1 (2017): 33–37. 10.3346/jkms.2017.32.1.33 PMC5143295 27914129

[17] F. Davatchi , S. Assaad‐Khalil , K. T. Calamia , et al., “The International Criteria for Behçet's Disease (ICBD): A Collaborative Study of 27 Countries on the Sensitivity and Specificity of the New Criteria,” Journal of the European Academy of Dermatology and Venereology 28, no. 3 (2014): 338–347. 10.1111/jdv.12107 23441863

[18] World Medical Association , “World Medical Association Declaration of Helsinki: Ethical Principles for Medical Research Involving Human Subjects,” Journal of the American Medical Association 310, no. 20 (2013): 2191–2194. 10.1001/jama.2013.281053 24141714

[19] Y. Kirino , G. Bertsias , Y. Ishigatsubo , et al., “Genome‐Wide Association Analysis Identifies New Susceptibility Loci for Behçet's Disease and Epistasis Between HLA‐B*51 and ERAP1,” Nature Genetics 45, no. 2 (2013): 202–207. 10.1038/ng.2520 PMC3810947 23291587

[20] H. Yazici , E. Seyahi , G. Hatemi , and Y. Yazici , “Behçet Syndrome: A Contemporary View,” Nature Reviews Rheumatology 14, no. 2 (2018): 107–119. 10.1038/nrrheum.2017.208 29296024

[21] I. Tugal‐Tutkun , “Behçet's Uveitis,” Middle East African Journal of Ophthalmology 16, no. 4 (October 2009): 219–224, 10.4103/0974-9233.58425. PMC2855662 20404988

