# Supplementary material for: Ethnic Differences in Clinical Manifestations of Behçet's Disease: A Retrospective Cohort Study of Turkic and Non‐Turkic Patients
Source: Immun Inflamm Dis. 2026 Nov 5;14(11):e70568. doi: 10.1002/iid3.70568 (PMC13737468; doi:10.1002/iid3.70568)
Supplement: Supplementary file 1 [file IID3-14-e70568-s001.docx]

**Supplementary Figure 1** Heatmap of conditional probabilities for major clinical features across latent classes identified by latent class analysis (LCA). Each cell represents the estimated probability of the presence of a given clinical feature within a specific latent class. Darker colors indicate higher conditional probabilities, and the numerical values correspond to the estimated probabilities. The identified classes (Class I-III) represent distinct clinical phenotypes based on patterns of organ involvement.
